# Thermal Upgrade of Enzymatically Synthesized Aliphatic and Aromatic Oligoesters

**DOI:** 10.3390/ma13020368

**Published:** 2020-01-13

**Authors:** James W. Comerford, Fergal P. Byrne, Simone Weinberger, Thomas J. Farmer, Georg M. Guebitz, Lucia Gardossi, Alessandro Pellis

**Affiliations:** 1 Green Chemistry Centre of Excellence, Department of Chemistry, University of York, Heslington, York YO10 5DD, UKfergal.byrne@york.ac.uk (F.P.B.); thomas.farmer@york.ac.uk (T.J.F.); 2 Department of Agrobiotechnology IFA-Tulln, Institute of Environmental Biotechnology, University of Natural Resources and Life Sciences, Konrad Lorenz Strasse 20, 3430 Tulln an der Donau, Austria; simone.weinberger@boku.ac.at (S.W.); guebitz@boku.ac.at (G.M.G.); 3 Division Enzymes & Polymers, Austrian Centre of Industrial Biotechnology, Konrad Lorenz Strasse 20, 3430 Tulln an der Donau, Austria; 4 Dipartimento di Scienze Chimiche e Farmaceutiche, Università degli Studi di Trieste, Piazzale Europa 1, 34127 Trieste, Italy; gardossi@units.it

**Keywords:** bio-based polyesters, enzymatic synthesis, polycondensation, thermal upgrade, metal-free synthesis, biocatalyzed process, solventless reactions

## Abstract

The enzymatic synthesis of polyesters in solventless systems is an environmentally friendly and sustainable method for synthetizing bio-derived materials. Despite the greenness of the technique, in most cases only short oligoesters are obtained, with limited practical applications or requiring further chemical processing for their elongation. In this work, we present a catalyst-free thermal upgrade of enzymatically synthesized oligoesters. Different aliphatic and aromatic oligoesters were synthesized using immobilized *Candida antarctica* lipase B (iCaLB) as the catalyst (70 °C, 24 h) yielding poly(1,4-butylene adipate) (PBA, M_w_ = 2200), poly(1,4-butylene isophthalate) (PBI, M_w_ = 1000), poly(1,4-butylene 2,5-furandicarboxylate) (PBF, M_w_ = 600), and poly(1,4-butylene 2,4-pyridinedicarboxylate) (PBP, M_w_ = 1000). These polyesters were successfully thermally treated to obtain an increase in M_w_ of 8.5, 2.6, 3.3, and 2.7 folds, respectively. This investigation focused on the most successful upgrade, poly(1,4-butylene adipate), then discussed the possible effect of di-ester monomers as compared to di-acids in the thermally driven polycondensation. The herein-described two-step synthesis method represents a practical and cost-effective way to synthesize higher-molecular-weight polymers without the use of toxic metal catalysts such as titanium(IV) *tert*-butoxide, tin(II) 2-ethylhexanoate, and in particular, antimony(IV) oxide. At the same time, the method allows for the extension of the number of reuses of the biocatalyst by preventing its exposure to extreme denaturating conditions.

## 1. Introduction

Due to their remarkable catalytic efficiency, enzymes are attractive and sustainable alternatives to toxic catalysts (such as antimony and titanium) commonly used in polycondensation and ring-opening polymerizations (such as tin). *Candida antarctica* lipase B (CaLB), a lipase isolated from a yeast, is the most frequently used enzyme for transesterification reactions due to its ability to work efficiently in low-water environments and in a wide range of organic solvents [1,2,3] at temperatures up to 100 °C [4,5]. The use of an enzyme catalyst for polyester synthesis is usually required when a high selectivity of the reaction is desired (e.g., the esterification of primary hydroxy groups only when glycerol is used as the diol) [6,7] and when temperature-sensitive moieties (such as itaconic acid, 1,4-cyclohexanedimethanol, and sorbitol) are used. Such monomers suffer from several side reaction such as C=C regioisomerization and cross-linking (itaconic acid) [8,9], thermal decomposition (1,4-cyclohexanedimethanol) [10], or dehydration (sorbitol) [11] under the harsh reaction conditions usually required by conventional chemical processes (t > 180 °C). Despite the high selectivity and the greenness of enzymatic synthesis, one of the main limitations of the enzymatically synthesized polyesters (relative to chemocatalytic equivalents) is the typically lower molecular weight of the isolated products. The industrial-scale enzymatic synthesis of polyesters was a challenge that was first tackled in the 1990s when Baxenden Chemicals (UK) transferred the general concept of enzyme-catalyzed condensation of polyols and diacids to a multi-kg scale, with the final goal of obtaining highly regular polymeric structures for coating and adhesive applications [12]. This plant is now decommissioned, and the development of new chemo-enzymatic synthesis technologies, which allow the synthesis of structure-controlled polymers with high molecular weights and low operational costs, remains a challenge.

To improve the increase in the molecular weights of enzymatically synthesized polyesters, several synthetic methods have been reported that use high-boiling solvents, which drive the reaction to completion and promote elongation of the polyester chain by stripping out the condensate byproduct (usually an alcohol or water) from the reaction system [2,3,11]. Unfortunately, the scale-up of such procedures is complicated due to the use of various solvents required for polymer isolation (diphenyl ether for the reaction, chloroform for the filtration step, and methanol for the precipitation of the polymer).

Notably, azeotropic water removal by aromatic solvents in the presence of acid catalysts favors cyclic ether formation due to diol cyclization in dilute systems [13,14]. The limitation of chain growth by diol cyclization is a common problem when reactions in solution are concerned [15], thus supporting the application of solventless conditions.

In an earlier study by the current authors, solvent-free polycondensation of adipic acid (AA) and 1,4-butanediol (BDO) was carried out in two steps, with the objective of promoting the elongation of the polyester chain while preserving the integrity of the immobilized biocatalyst [12]. A first step was performed at 50 °C at atmospheric pressure in the presence of the enzyme. The biocatalyst was then removed, and the second step was initiated by increasing the temperature to 80 °C under reduced pressure (70 mbar). Reduced pressure was necessary as uncatalyzed polycondensations of alcohols with carboxylic acids have low equilibrium constants (generally K_C_ < 10), so water, obtained as a reaction byproduct, had to be efficiently removed from the reaction mixture in order to obtain a reasonable degree of polymerization [16]. Under such conditions, modest progress of the polycondensation was observed after 72 h. The removal of the biocatalyst after the synthesis of oligomers is appealing, since the viscosity increases as the reaction proceeds, and the recovery of the biocatalyst becomes significantly more challenging. The same authors reported a more striking elongation of the polyester chain by performing the two-step procedure in a turbo-reactor under thin-film conditions and atmospheric pressure [17]. The first biocatalyzed step at 60 °C yielded oligomers with an average molecular weight (M_n_) of ~1800, whereas the second catalyst-free step at 90 °C led to an improvement of M_n_ up to 2900. It must be noted that the polyesterification in the absence of the biocatalyst is already a well-known reaction, with the mechanism and kinetics of the self-catalyzed polyesterification reactions of AA and diols that have been reported before [18,19]. Briefly, protons dissociate from the diacid molecules but continue to weakly coordinate with the diacid molecules. This suggests that the self-catalyzed polyesterification is promoted by such coordinating protons [20].

**Scheme 1:**
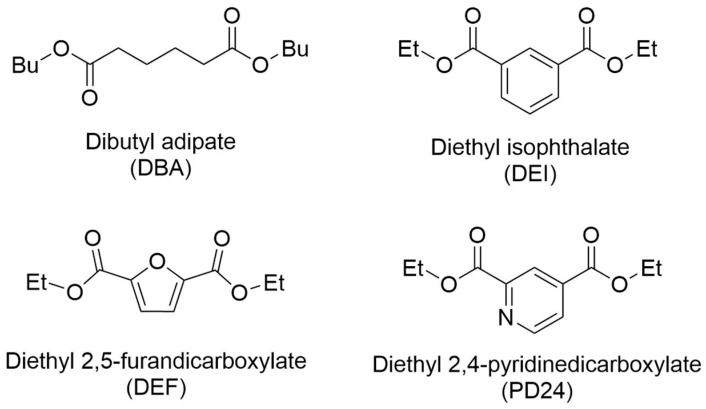
Aliphatic and aromatic diesters used as starting materials for the enzymatic synthesis of bio-based and petroleum-based oligoesters. All polyesters in this work were synthesized from these diesters and from 1,4-butanediol (1,4-BDO) as the aliphatic, linear diol.

However, no detailed study on the catalyst-free polycondensation of di-esters has been reported so far. The evidence above prompted us to explore how to exploit the two-step procedures for the solventless synthesis of both aliphatic and aromatic short oligomers, using adipate, isophthalate, furan, and pyridine diesters as models (Scheme 1). In this way, the reaction products can be easily separated from the biocatalyst in a single filtration step, allowing the obtained oligomers to be further processed via a catalyst-free thermal upgrade to oligomers and polymers of higher molecular weights which are suitable for a whole series of applications.

## 2. Results and Discussion

### 2.1. Enzymatic Synthesis of Aliphatic and Aromatic Oligoesters

The solventless, enzyme-catalyzed synthesis of short aliphatic and aromatic oligoesters was conducted following previously published procedures [2,21,22]. Poly(1,4-butylene adipate) (PBA), poly(1,4-butylene isophthalate) (PBI), poly(1,4-butylene 2,5-furandicarboxylate) (PBF), and poly(1,4-butylene 2,4-pyridinedicarboxylate) (PBP) were successfully synthesized and their properties are shown in Table 1 in comparison to the same polyesters synthesized at 85 °C without enzymes [2,23].

**Scheme 2:**
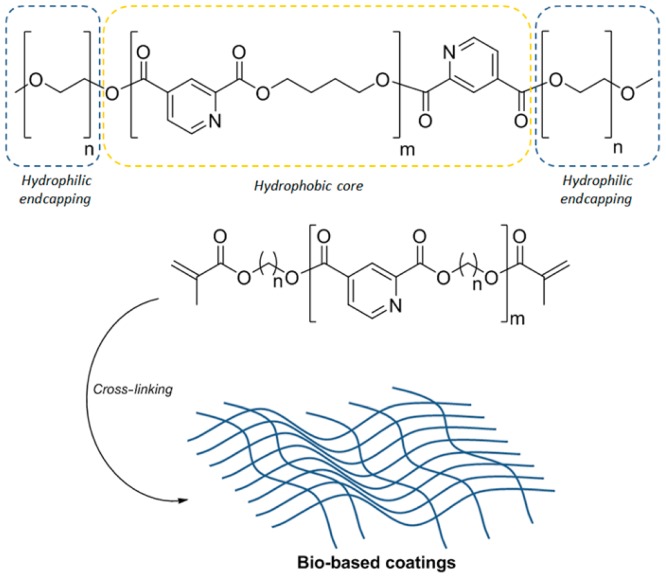
Potential use of the enzymatically synthesized oligoesters using a reaction temperature of 85 °C. Synthesis of amphiphile polyesters (**top**) and coupling with acrylates for the formation of bio-based coatings upon cross-linking (**bottom**). The utilization of the enzymatically synthesized oligoesters will be presented in a future full paper.

In addition, the reactions were conducted at a lower reaction temperature compared to previous works on the topic, with the ambition of improving biocatalyst recyclability. Despite most studies in the literature reporting the synthesis of polyesters using CaLB at temperatures around 85 °C [2,23], it is widely known that lower reaction temperatures ensure the preservation of the enzyme’s activity over repeated reaction cycles [12]. With the immobilized biocatalyst being the most expensive component of the system, its recyclability is a key parameter to consider when conducting polycondensation reactions [24]. The synthesized oligoesters can have several applications upon end chain endcapping and cross-linking as detailed in Scheme 2.

### 2.2. Catalyst-Free Thermal Upgrade of the Enzymatically Synthesized Oligoesters

After the removal of the biocatalyst (via filtration) and of the work-up solvent (via rotary evaporation), the enzymatically synthesized oligoesters were subjected to temperatures ranging from 140 to 180 °C in three different conditions: air, under a 1 mbar vacuum, and under an inert N_2_ atmosphere.

**Figure 1:**
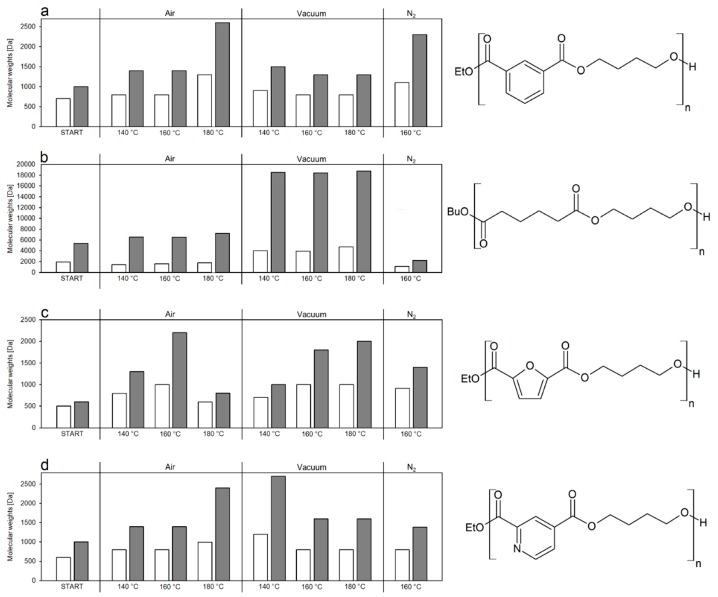
Molecular weight analysis of the catalyst-free thermal upgrade of the enzymatically synthesized oligoesters. (**a**) Poly(1,4-butylene isophthalate) (PBI), (**b**) poly(1,4-butylene adipate) (PBA) synthesized starting from dibutyl adipate (DBA), (**c**) poly(1,4-butylene 2,5-furandicarboxylate) (PBF), and (**d**) poly(1,4-butylene 2,4-pyridinedicarboxylate) (PBP). White bars = Number-Average Molecular Weight (M_n_); grey bars = Weight-Average Molecular Weight (M_w_).

All performed thermal upgrades were successful (Figure 1) and led to an increase in the degree of polymerization (as evidenced by the increased number-average molecular weight (M_n_) and weight-average molecular weight (M_w_)) of the enzymatically synthesized oligoesters.

The enzymatically synthesized PBI’s weight-average molecular weight increased 2.6 times (M_w_ increasing from 1000 to 2600 Da) when using 180 °C and an air atmosphere, and 2.3 times (M_w_ increasing from 1000 to 2300 Da) when using 160 °C and a N_2_ atmosphere. All other tested conditions also led to slightly higher molecular weights, but the increases were less pronounced than the two described above (Figure 1a). For diethyl isophthalate (DEI) as the diester, it seems that a higher reaction temperature leads to a better chain elongation, whereas applying a vacuum does not have any major influence on the oligomers. DEI is known to be a temperature-stable molecule (usually polymerized chemo-catalytically at 260 °C [25]) and all produced materials result in white-colored polymers (see Supplementary Materials Figure S1). Upgrade of the aliphatic oligoester PBA was the most successful in this study, since the weight-average molecular weight of the initial oligomers increased from 5400 Da to over 18,000 Da when conducting the upgrades under vacuum. Of note is that the thermal upgrade in the air is not feasible since the reaction mixture quickly changes color from white (starting oligomers after enzymatic synthesis, see Supplementary Materials Figure S2) to yellowish and dark brown when reaction temperatures above 140 °C are used (see Supplementary Materials Figure S3). The formation of colored reaction products, together with the almost-absent increase of the oligomer’s molecular weight, is a clear sign that temperature-related side reactions occur with the consequent partial degradation of parts of the oligomers.

In conclusion, the fact that the thermal treatment of polyesters in vacuum resulted in a successful upgrade of the oligomers is in line with previous results reported for the synthesis of PBA starting from adipic acid [12,17]. It is known that the self-catalyzed polyesterifications follow third-order kinetics, with a second-order dependence on the carboxyl group concentration, and a first-order dependence on the hydroxyl group concentration. However, also in the case of the alcoholysis of esters, once a mixture of short oligomers is formed, the polycondensation proceeds without the need for the external addition of the catalyst, thus suggesting the role of entropic factors in driving the process. Indeed, studies of the kinetics of polycondensation reported models describing the increase of the reaction order as the esterification proceeds, demonstrating that by increasing the M_n_ the reaction can proceed at lower temperatures [26].

The present data show that furan and pyridine moieties have similar temperature sensitivities, which led to rapid coloration of the reaction products (and a slight increase of the molecular weights) when the thermal upgrade was conducted in air (see Supplementary Materials Figures S4 and S5). Despite an increase of the molecular weights for the reactions conducted in air, the best results in terms of chain extension were obtained for the reactions under vacuum that led to a 3.3 and a 2.7 times higher M_w_ for PBF and PBP, respectively, without leading to significant changes in the coloration of the reaction product.

## 3. Materials and Methods

### 3.1. Materials and Enzymes

1,4-butanediol (1,4-BDO) was purchased from Alfa Aesar (Haverhill, MA, USA). Dimethyl adipate (DMA) was purchased from Acros Organics (Waltham, MA, USA). Diethyl isophthalate (DEI) was purchased from Syntree Inc. (Hangzhou, China). Diethyl-2,5-furandicarboxylate (DEF) and diethyl pyridine-2,4-dicarboxylate (PD24) were purchased from Carbosynth (Compton, UK). Dibutyl adipate (DBA) and all other chemicals and solvents were purchased from Sigma-Aldrich (St. Louis, MI, USA) and used without further purification. Lipase B from *Candida antarctica* (CaLB) immobilized onto methacrylic resin was also purchased from Sigma-Aldrich (product code L4777, also known as N435). The enzyme was dried under vacuum for 48 h at 25 °C and stored in a desiccator prior to use.

### 3.2. Enzymatic Synthesis of Aliphatic and Aromatic Oligoesters

Dicarboxylic acid ester (A, 0.006 mol) and linear diol (B, 0.006 mol) (diester/diol ratio = 1.0:1.0) were added in a 25 mL round-bottom flask. The monomer’s mixture was stirred at 50 °C until a homogeneous melt was obtained. Then 10% w w^−1^ (calculated on the total amount of the monomers) of immobilized CaLB was added to the monomer’s mixture, and the reaction was run for 6 h at a pressure of 1000 mbar. A vacuum of 20 mbar was subsequently applied for an additional 18 h, maintaining the reaction temperature at 50 °C (total reaction time of 24 h). The reaction products were recovered by adding tetrahydrofuran (THF) (aliphatic polyesters) or dichloromethane (DCM) (aromatic polyesters) to dissolve the solid reaction products. The biocatalyst was then removed via a filtration step and the solvent evaporated via rotary evaporation. The polymers were then characterized without additional purification steps.

### 3.3. Catalyst-Free Thermal Upgrade of Oligomers

For the thermal upgrades, 30 mg of enzymatically synthesized oligomers were added in an 8 mL flat-bottomed vial, together with a magnetic stirring bar. The vial was then heated up to 140, 160, or 180 °C under the appropriate N_2_/air atmosphere or vacuum, and left stirring at 400 rpm for 24 h. After the upgrade, the reaction mixture was analyzed without prior purification steps.

### 3.4. Nuclear Magnetic Resonance (NMR) Spectroscopy

^1^H-NMR analysis was performed on a JEOL JNM-ECS400A spectrometer (JEOL, Peabody, MA, USA) at a frequency of 400 MHz. CDCl_3_ was used as the NMR solvent for all synthesized polymers. Please see Supplementary Information, Figures S6–S10 for the ^1^H-NMR spectra of the PBA polymers, Figures S11–S15 for the ^1^H-NMR spectra of the PBI polymers, Figures S16–S20 for the ^1^H-NMR spectra of the PBF polymers and Figures S21–S25 for the ^1^H-NMR spectra of the PBP polymers.

### 3.5. Gel Permeation Chromatography (GPC)

Samples were dissolved in CHCl_3_ (final concentration ~1 mg mL^−1^) and filtered through a cotton filter into an HPLC vial. Gel permeation chromatography was carried out at a temperature of 30 °C on an Agilent Technologies HPLC System (Agilent Technologies 1260 Infinity, Agilent Technologies, Santa Clara, CA, USA) equipped with a 17369 6.0 mm ID × 40 mm L HHR-H, 5 μm Guard column, and an 18055 7.8 mm ID × 300 mm L GMHHR-N, 5 μm TSKgel liquid chromatography column (Tosoh Bioscience, Tessenderlo, Belgium). Mobile phase was CHCl_3_ at a flow rate of 1 mL min^−1^. A refractive index detector (Agilent Technologies G1362A) was employed for the detection of the obtained oligomers. A linear polystyrene-calibration standard curve was used for determining the molecular weights of the synthesized polymers.

### 3.6. Matrix-Assisted Laser Desorption Ionization (MALDI)

MALDI-TOF MS analyses were carried out using a Bruker Solarix-XR FTICR mass spectrometer and the relative software package for the acquisition and the processing of the data. The used acceleration voltage was 25 kV. DCTB was selected as the matrix and KTFA as the ionization agent. Then 10 μL of sample were mixed with 10 μL of matrix solution (40 mg mL^−1^ DCTB in THF) and 3 μL of KTFA (5 mg mL^−1^). The mixture (0.3 μL) was applied on the plate. The measurement of all samples was conducted in positive mode. The detector was set in reflector mode.

## 4. Conclusions

The potential of thermal treatments for the chain elongation of enzymatically synthesized polyesters was studied. *Candida antarctica* lipase B was used for the synthesis of various aliphatic and aromatic oligoesters, namely poly(1,4-butylene adipate) (M_n_ = 1000 g mol^−1^, M_w_ = 2200 g mol^−1^), poly(1,4-butylene isophthalate) (M_n_ = 700 mol^−1^, M_w_ = 1000 g mol^−1^), poly(1,4-butylene 2,5-furandicarboxylate) (M_n_ = 500 g mol^−1^, M_w_ = 600 g mol^−1^), and poly(1,4-butylene 2,4-pyridinedicarboxylate) (M_n_ = 600 g mol^−1^, M_w_ = 1000 g mol^−1^). A thermal, catalyst-free treatment of these oligoesters was performed, yielding polymers having, respectively, 8.5, 2.6, 3.3, and 2.7 times higher weight-average molecular weights than the initial oligomers. This two-step strategy allows the greening of high molecular weight bio-based polyester synthesis, since no solvents or toxic metal and acid catalysts are used in the process.

## Figures and Tables

**Table 1:** Enzymatic synthesis of aliphatic and aromatic oligoesters at various temperatures in solventless reaction systems.

Polymer	Synthesis t (°C)	Monomers Conversion * (%)	M_n_^+^ (Da)	M_w_^+^ (Da)	Đ ^+^	Reference
PBA ^a^	50	96	1900	5400	2.89	This work
	85	96	4200	6400	1.53	[23]
PBI	50	81	700	1000	1.41	This work
	85	84	900	1500	1.62	[2]
PBF	50	78	500	600	1.19	This work
	85	79	600	900	1.34	[2]
PBP	50	80	600	1000	1.52	This work
	85	82	800	1400	1.65	[2]

* Calculated via ^1^H-NMR; ^+^ Calculated via GPC; ^a^ Synthesized using dibutyl adipate (DBA) as the aliphatic diester.

## Supplementary Materials

The following are available online at https://www.mdpi.com/1996-1944/13/2/368/s1, Figure S1. Thermal upgrade of poly(1,4-butylene isophthalate) (PBI) conducted in air at 140 °C (left), 160 °C (centre), and 180 °C (right); Figure S2. Enzymatically synthesized poly(1,4-butylene adipate) (PBA) before conducting any thermal upgrade; Figure S3. Thermal upgrade of poly(1,4-butylene adipate) (PBA) conducted in air at 140 °C (left), 160 °C (centre), and 180 °C (right); Figure S4. Thermal upgrade of poly(1,4-butylene 2,5-furanoate) (PBF) conducted in air at 140 °C (left), 160 °C (centre), and 180 °C (right); Figure S5. Thermal upgrade of poly(1,4-butylene 2,4-pyridinoate) (PBP) conducted in air at 140 °C (left), 160 °C (centre), and 180 °C (right); Figure S6. ^1^H-NMR analysis of poly(1,4-butylene adipate) after the initial, solventless enzymatic synthesis step; Figure S7. ^1^H-NMR analysis of poly(1,4-butylene adipate) after the thermal upgrade conducted at 150 °C under vacuum; Figure S8. ^1^H-NMR analysis of poly(1,4-butylene adipate) after the thermal upgrade conducted at 140 °C under air; Figure S9. ^1^H-NMR analysis of poly(1,4-butylene adipate) after the thermal upgrade conducted at 160 °C under air; Figure S10. ^1^H-NMR analysis of poly(1,4-butylene adipate) after the thermal upgrade conducted at 180 °C under air; Figure S11. ^1^H-NMR analysis of poly(1,4-butylene isophthalate) after the initial, solventless enzymatic synthesis step; Figure S12. ^1^H-NMR analysis of poly(1,4-butylene isophthalate) after the thermal upgrade conducted at 150 °C under vacuum; Figure S13. ^1^H-NMR analysis of poly(1,4-butylene isophthalate) after the thermal upgrade conducted at 140 °C under air; Figure S14. ^1^H-NMR analysis of poly(1,4-butylene isophthalate) after the thermal upgrade conducted at 160 °C under air; Figure S15. ^1^H-NMR analysis of poly(1,4-butylene isophthalate) after the thermal upgrade conducted at 180 °C under air; Figure S16. ^1^H-NMR analysis of poly(1,4-butylene 2,5-furanoate) after the initial, solventless enzymatic synthesis step; Figure S17. ^1^H-NMR analysis of poly(1,4-butylene 2,5-furanoate) after the thermal upgrade conducted at 150 °C under vacuum; Figure S18. ^1^H-NMR analysis of poly(1,4-butylene 2,5-furanoate) after the thermal upgrade conducted at 140 °C under air; Figure S19. ^1^H-NMR analysis of poly(1,4-butylene 2,5-furanoate) after the thermal upgrade conducted at 160 °C under air; Figure S20. ^1^H-NMR analysis of poly(1,4-butylene 2,5-furanoate) after the thermal upgrade conducted at 180 °C under air; Figure S21. ^1^H-NMR analysis of poly(1,4-butylene 2,4-pyridinedicarboxylate) after the initial, solventless enzymatic synthesis step; Figure S22. ^1^H-NMR analysis of poly(1,4-butylene 2,4-pyridinedicarboxylate) after the thermal upgrade conducted at 150 °C under vacuum; Figure S23. ^1^H-NMR analysis of poly(1,4-butylene 2,4-pyridinedicarboxylate) after the thermal upgrade conducted at 140 °C under air; Figure S24. ^1^H-NMR analysis of poly(1,4-butylene 2,4-pyridinedicarboxylate) after the thermal upgrade conducted at 160 °C under air; Figure S25. ^1^H-NMR analysis of poly(1,4-butylene 2,4-pyridinedicarboxylate) after the thermal upgrade conducted at 180 °C under air.
