# Supplementary material for: Thermal Upgrade of Enzymatically Synthesized Aliphatic and Aromatic Oligoesters
Source: Materials (Basel). 2020 Jan 13;13(2):368. doi: 10.3390/ma13020368 (PMC7013642; doi:10.3390/ma13020368)
Supplement: Supplementary file 1 [file materials-13-00368-s001.pdf]

materials

Communication

Thermal Upgrade of Enzymatically Synthesized Aliphatic and Aromatic Oligoesters

James W. Comerford [1], Fergal P. Byrne [1], Simone Weinberger [2], Thomas J. Farmer [1], Georg M. Guebitz [2,3], Lucia Gardossi [4] and Alessandro Pellis [1,2,*]

1 Green Chemistry Centre of Excellence, Department of Chemistry, University of York, Heslington, York YO10 5DD, UK; james.comerford@york.ac.uk (J.M.C.); fergal.byrne@york.ac.uk (F.P.B.); thomas.farmer@york.ac.uk (T.J.F.)
2 Department of Agrobiotechnology IFA-Tulln, Institute of Environmental Biotechnology, University of Natural Resources and Life Sciences, Konrad Lorenz Strasse 20, Tulln an der Donau 3430, Austria; simone.weinberger@boku.ac.at (S.W.); guebitz@boku.ac.at (G.M.G.)
3 Division Enzymes & Polymers, Austrian Centre of Industrial Biotechnology, Konrad Lorenz Strasse 20, Tulln an der Donau 3430, Austria
4 Dipartimento di Scienze Chimiche e Farmaceutiche, Università degli Studi di Trieste, Piazzale Europa 1, 34127 Trieste, Italia; gardossi@units.it
* Correspondence: alessandro.pellis@boku.ac.at or alessandro.pellis@gmail.com

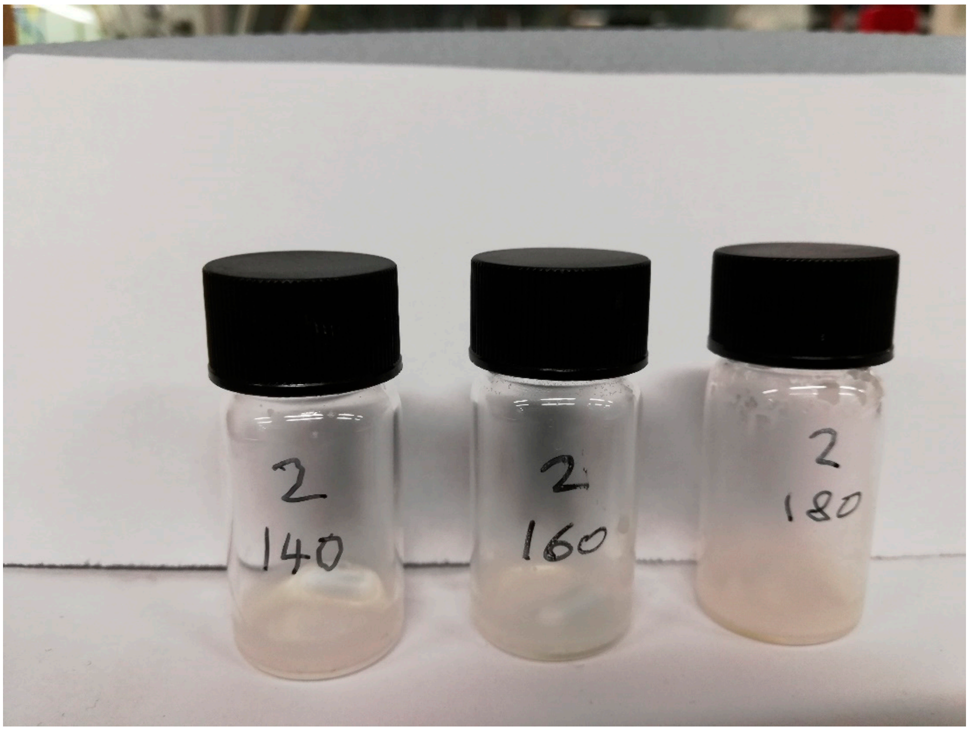

Figure S1. Thermal upgrade of poly(1,4-butylene isophthalate) (PBI) conducted in air at 140 °C (left), 160 °C (centre), and 180 °C (right).

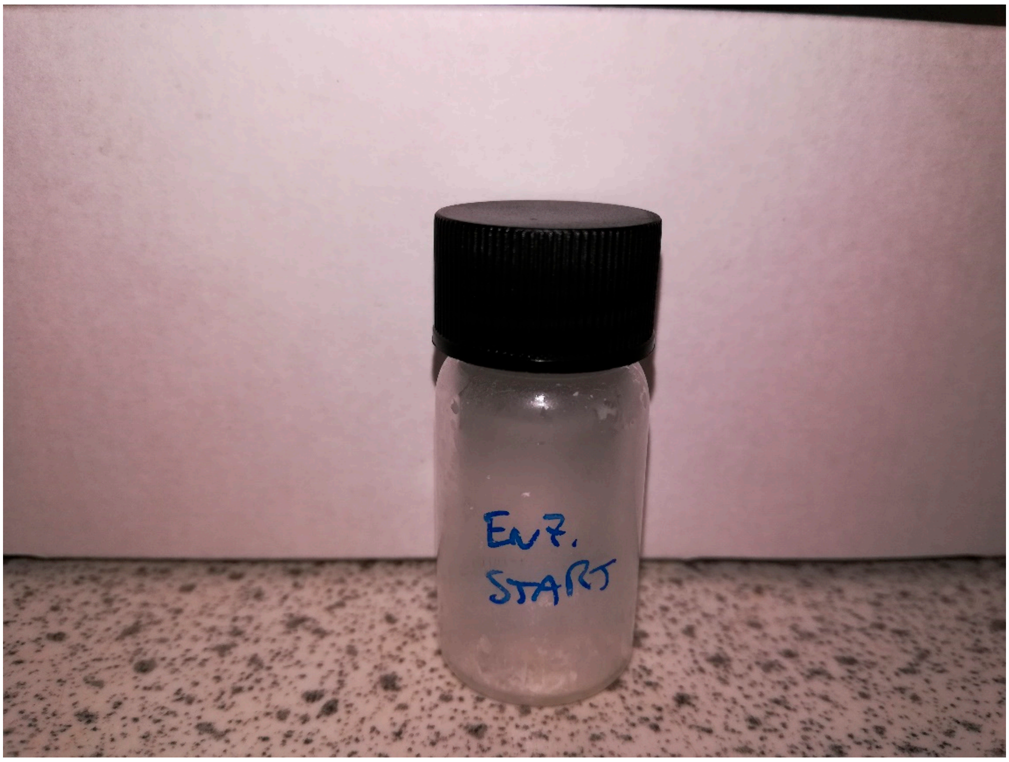

Figure S2. Enzymatically synthesized poly(1,4-butylene adipate) (PBA) before conducting any thermal upgrade.

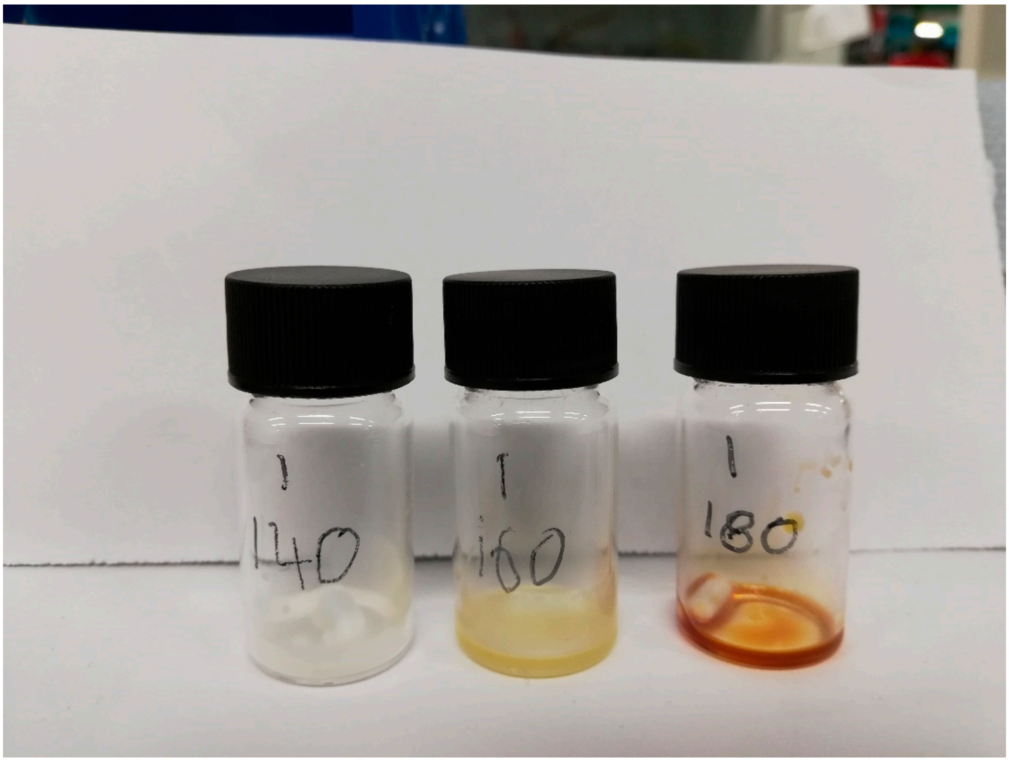

Figure S3. Thermal upgrade of poly(1,4-butylene adipate) (PBA) conducted in air at 140 °C (left), 160 °C (centre), and 180 °C (right).

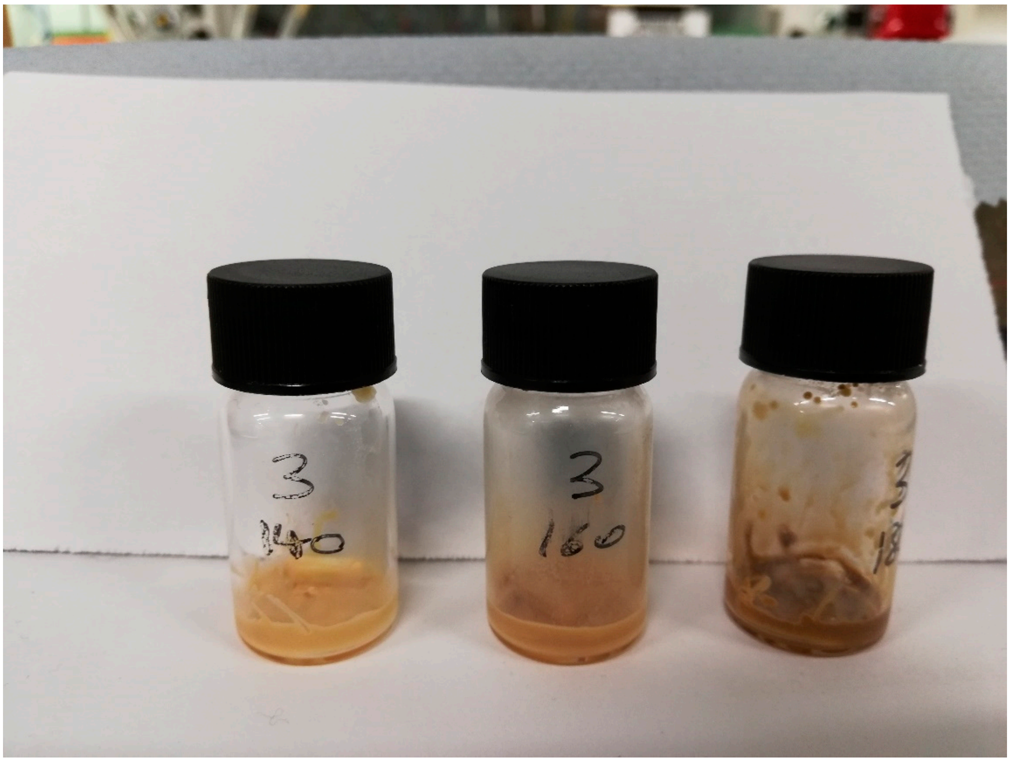

Figure S4. Thermal upgrade of poly(1,4-butylene 2,5-furanoate) (PBF) conducted in air at 140 °C (left), 160 °C (centre), and 180 °C (right).

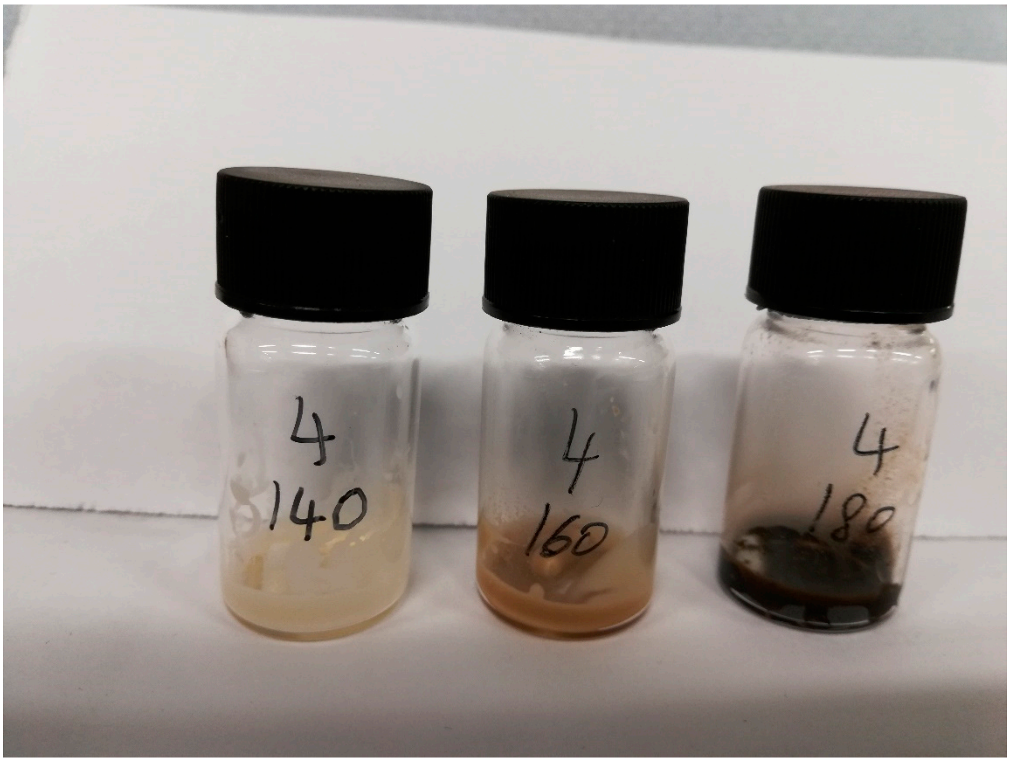

Figure S5. Thermal upgrade of poly(1,4-butylene 2,4-pyridinoate) (PBP) conducted in air at 140 °C (left), 160 °C (centre), and 180 °C (right).

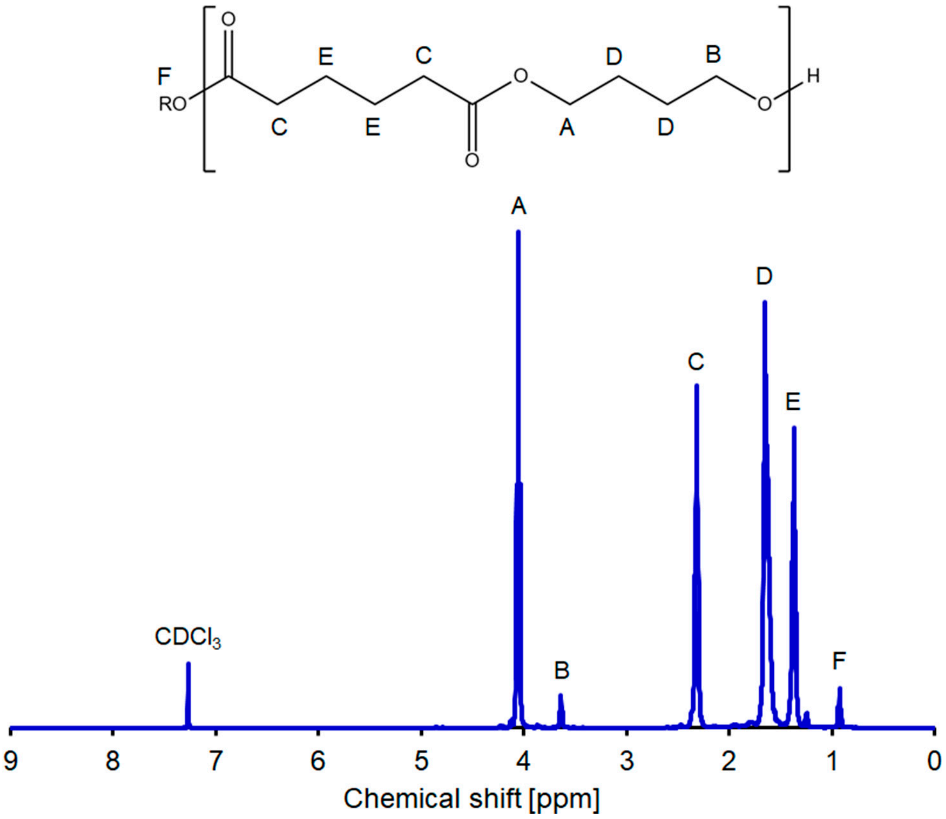

Figure S6. ^{1}H-NMR analysis of poly(1,4-butylene adipate) after the initial, solventless enzymatic synthesis step.

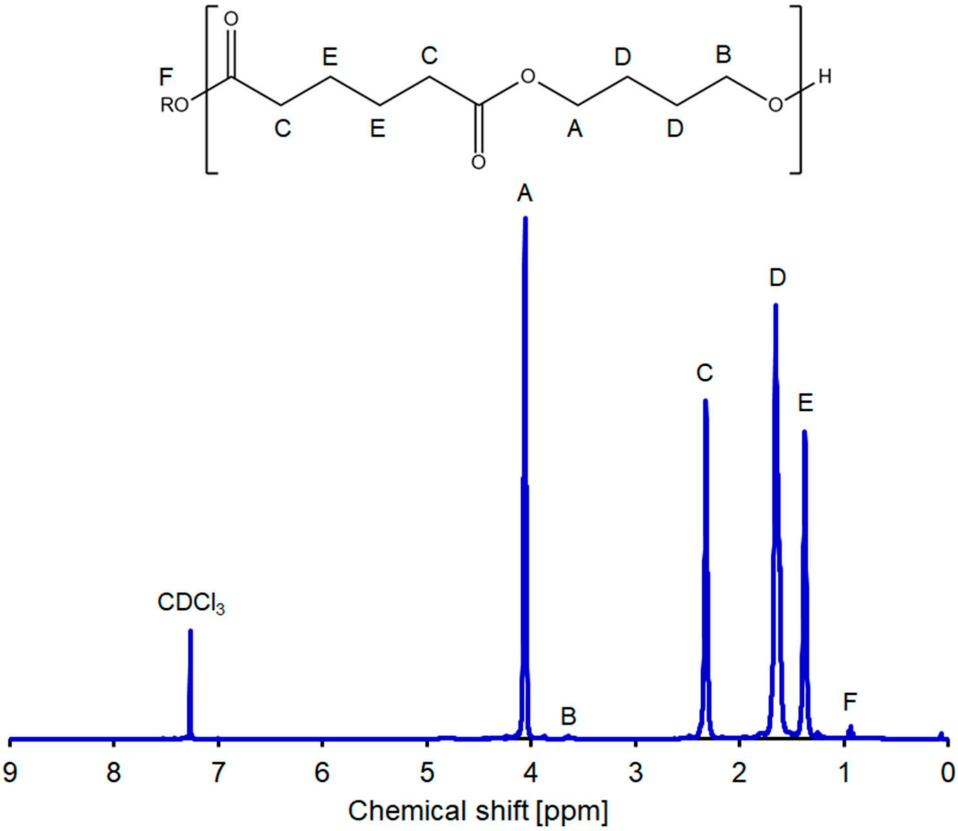

Figure S7. ^{1}H-NMR analysis of poly(1,4-butylene adipate) after the thermal upgrade conducted at 150 °C under vacuum.

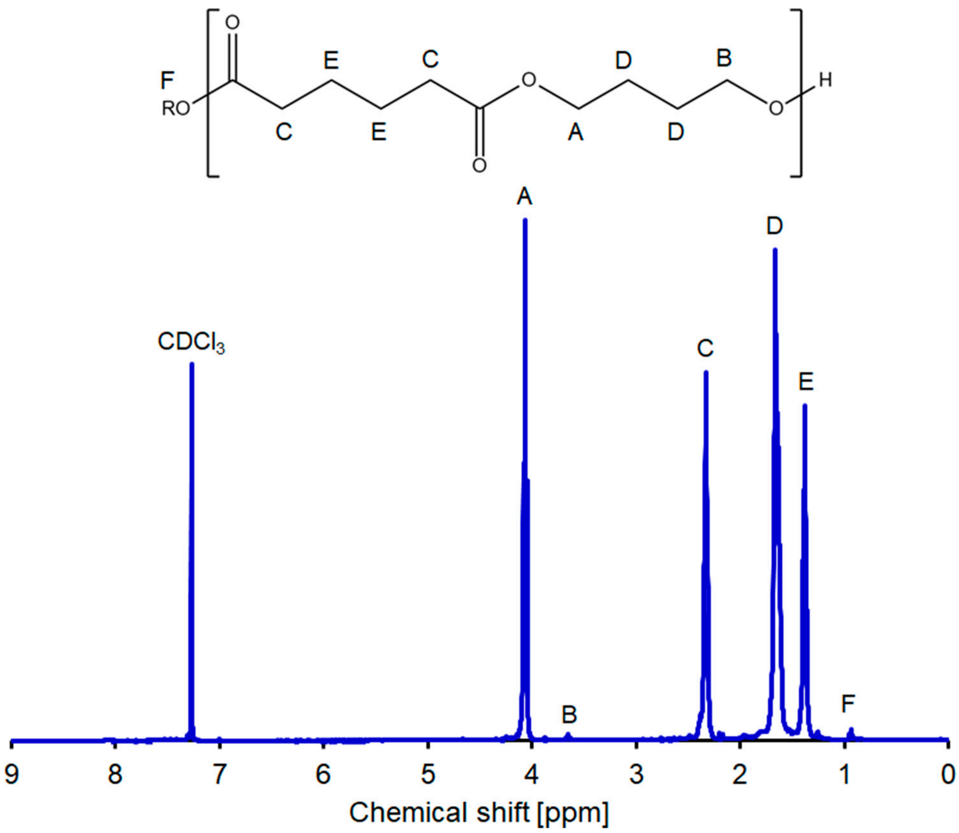

Figure S8. ^{1}H-NMR analysis of poly(1,4-butylene adipate) after the thermal upgrade conducted at 140 °C under air.

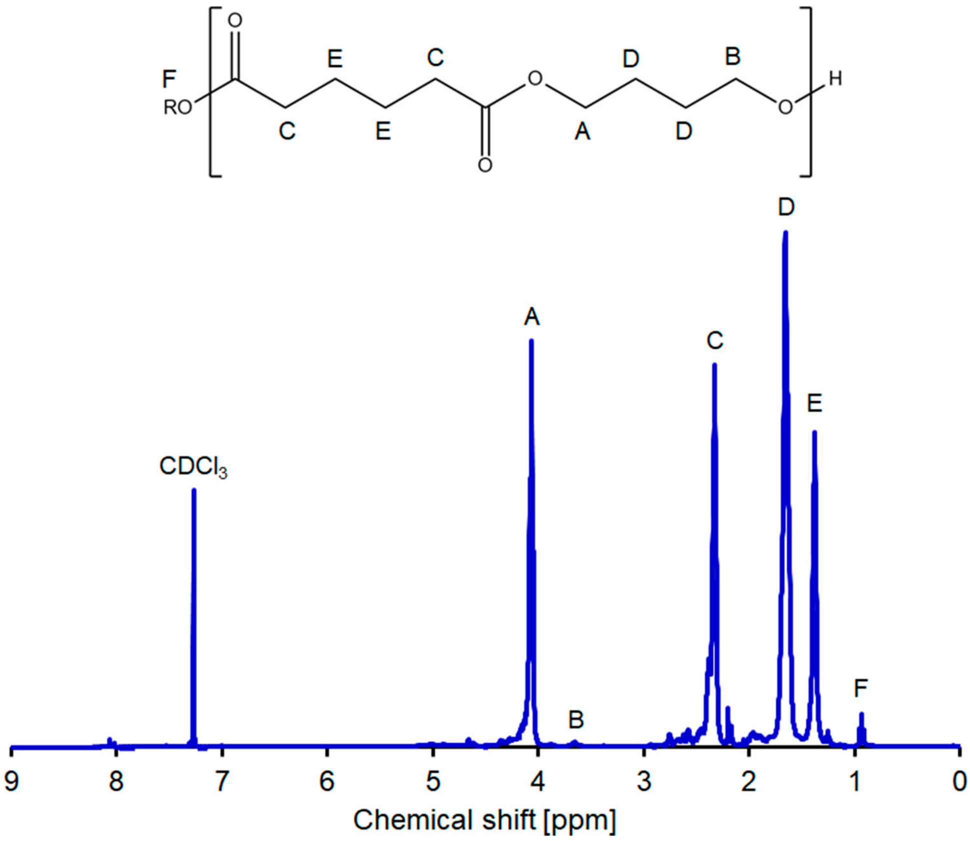

Figure S9. ^{1}H-NMR analysis of poly(1,4-butylene adipate) after the thermal upgrade conducted at 160 °C under air.

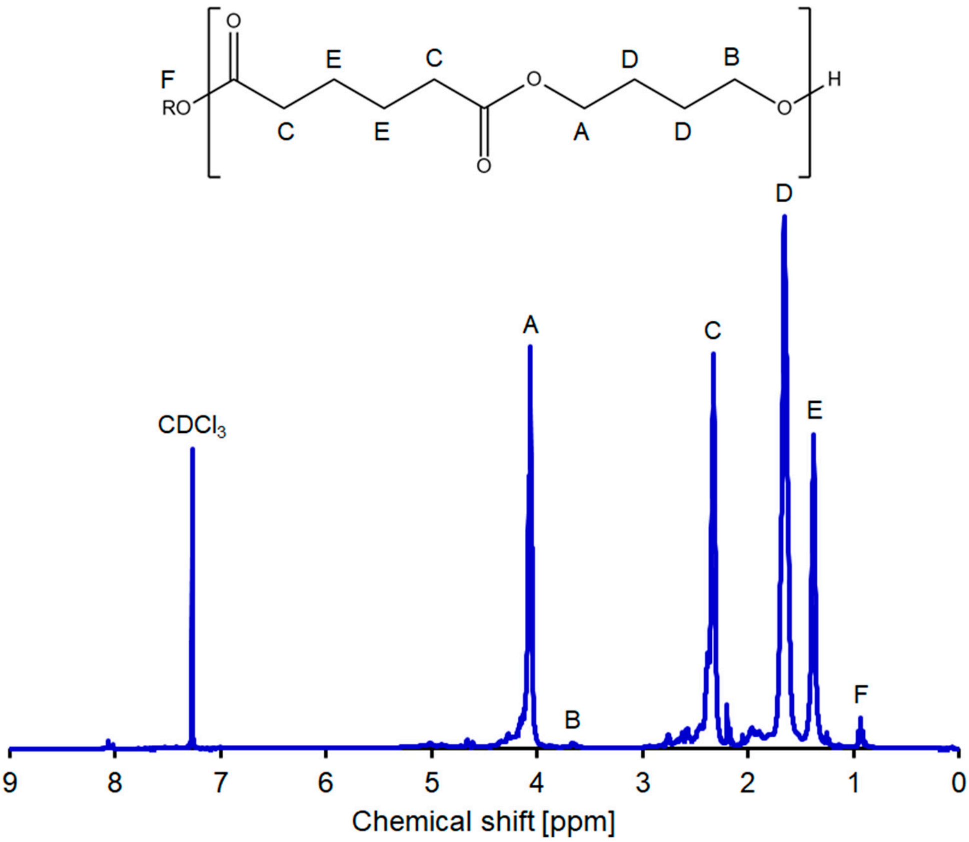

Figure S10. ¹H-NMR analysis of poly(1,4-butylene adipate) after the thermal upgrade conducted at 180 °C under air.

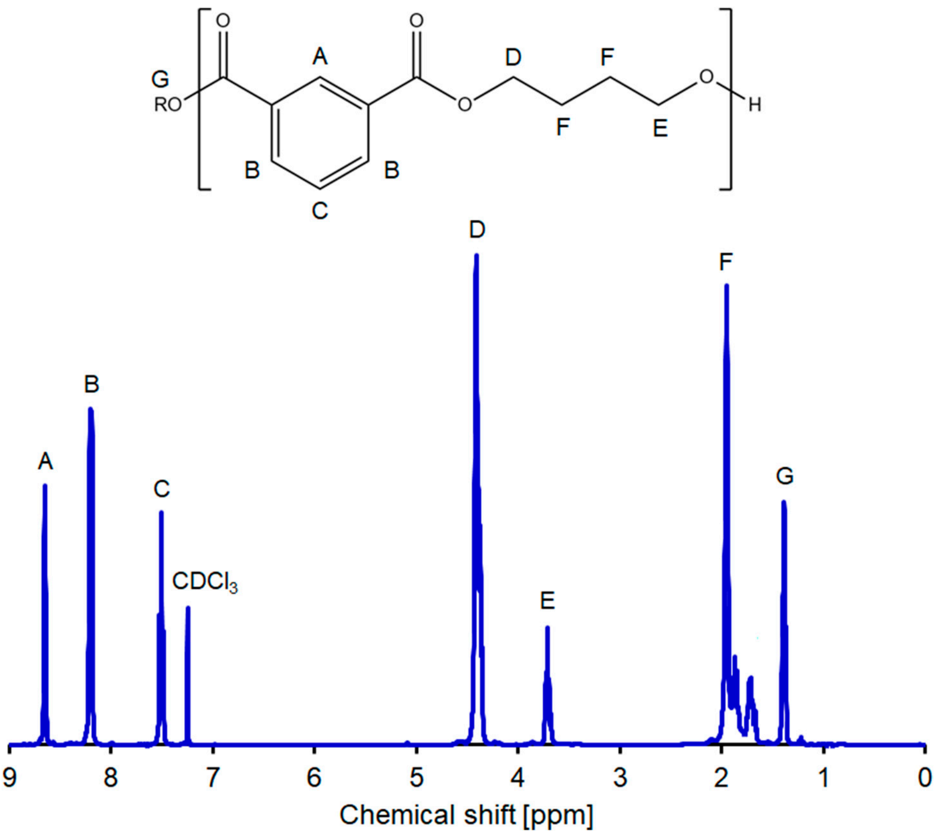

Figure S11. ¹H-NMR analysis of poly(1,4-butylene isophthalate) after the initial, solventless enzymatic synthesis step.

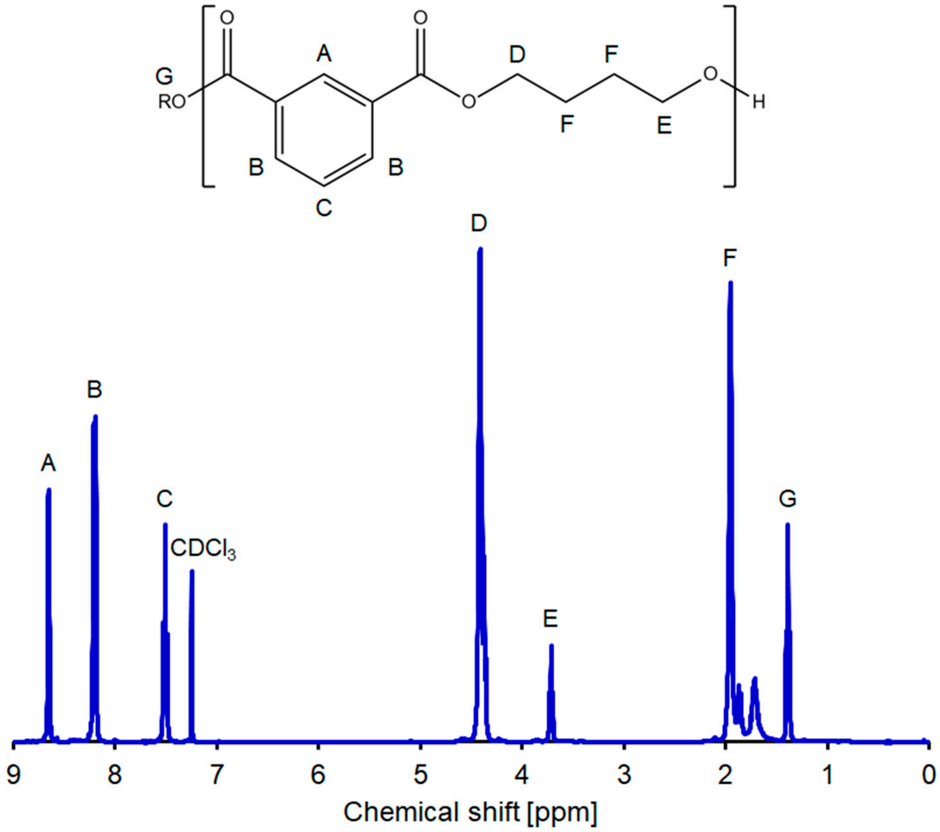

Figure S12. ^{1}H-NMR analysis of poly(1,4-butylene isophthalate) after the thermal upgrade conducted at 150 °C under vacuum.

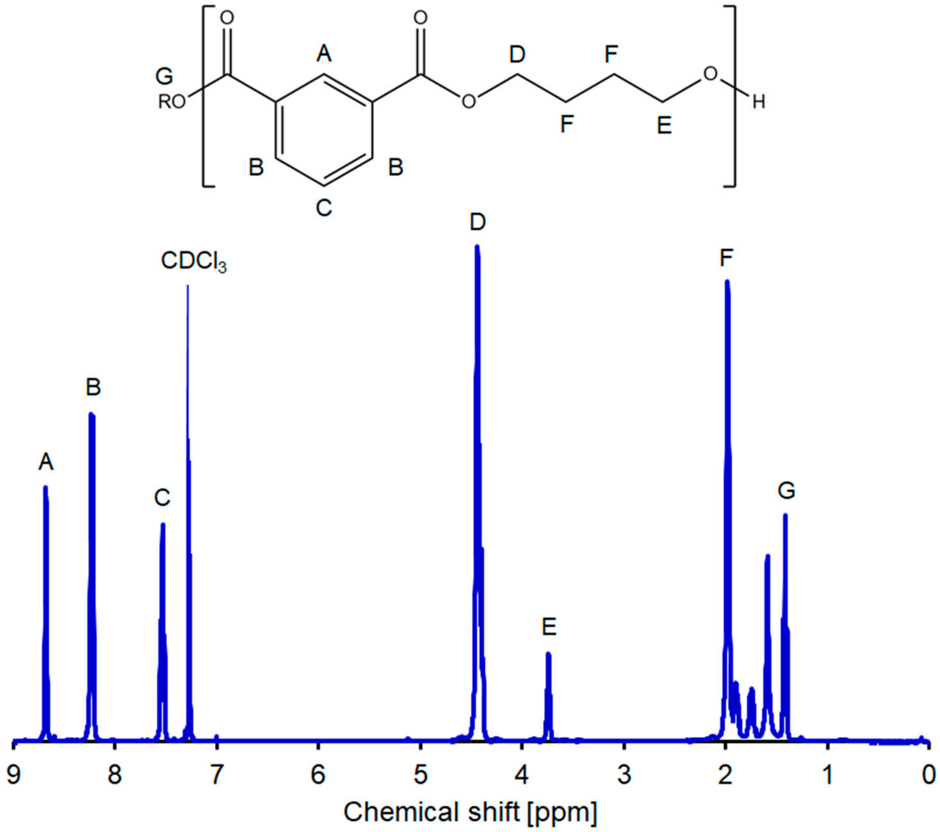

Figure S13. ^{1}H-NMR analysis of poly(1,4-butylene isophthalate) after the thermal upgrade conducted at 140 °C under air.

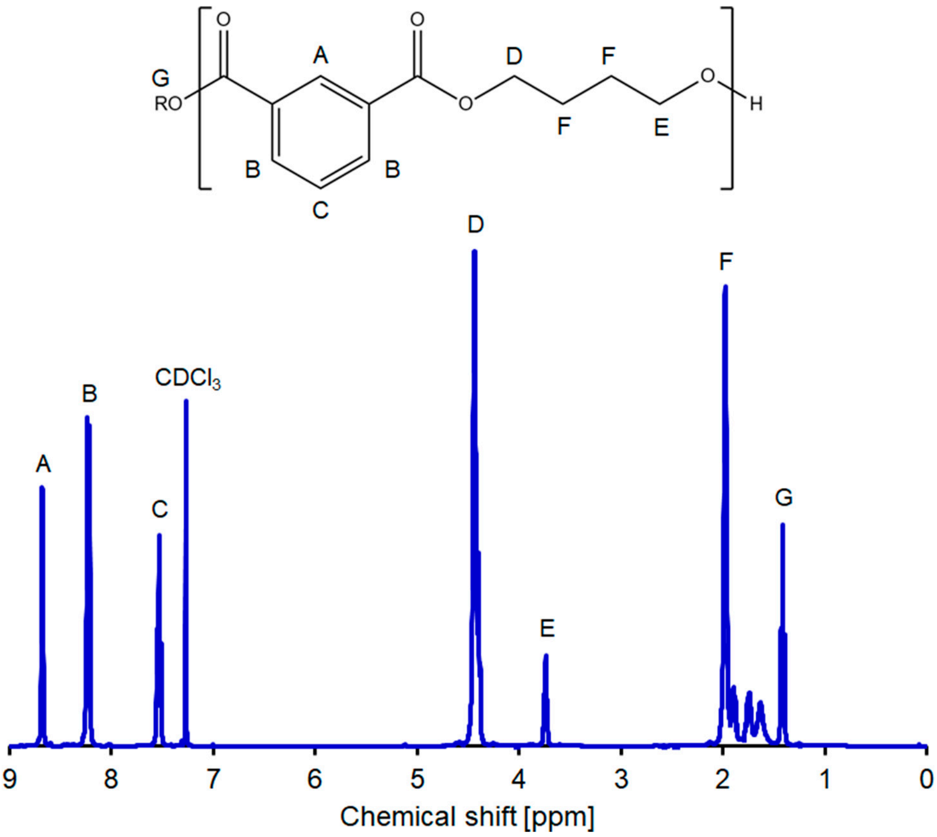

Figure S14. ^{1}H-NMR analysis of poly(1,4-butylene isophthalate) after the thermal upgrade conducted at 160 °C under air.

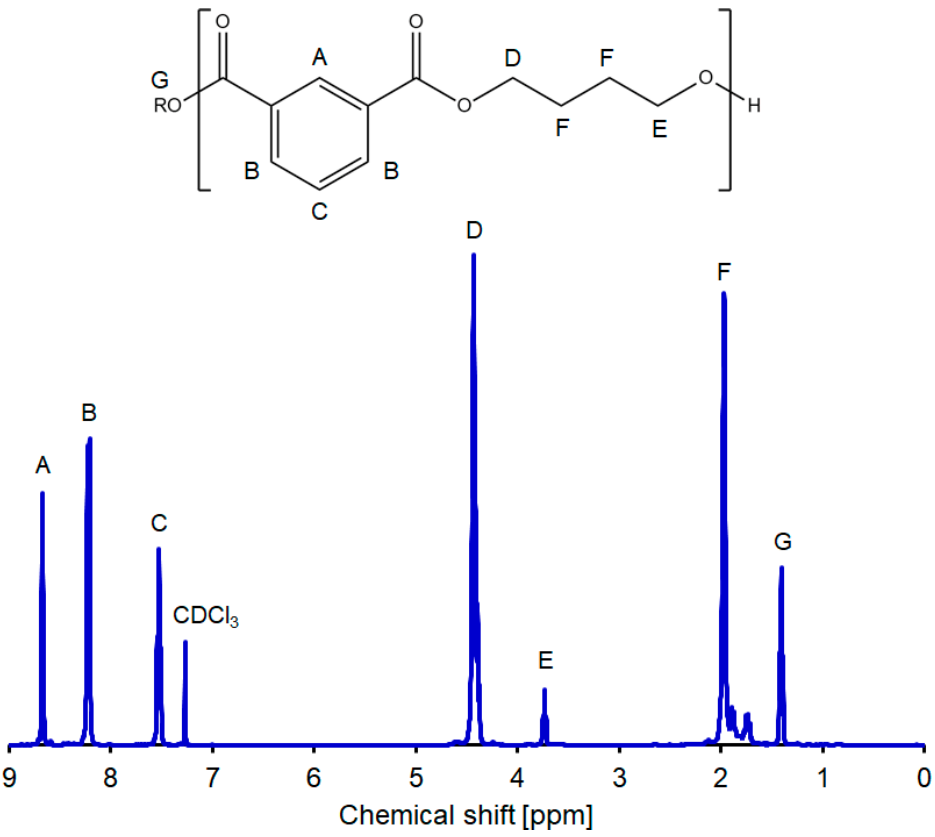

Figure S15. ^{1}H-NMR analysis of poly(1,4-butylene isophthalate) after the thermal upgrade conducted at 180 °C under air.

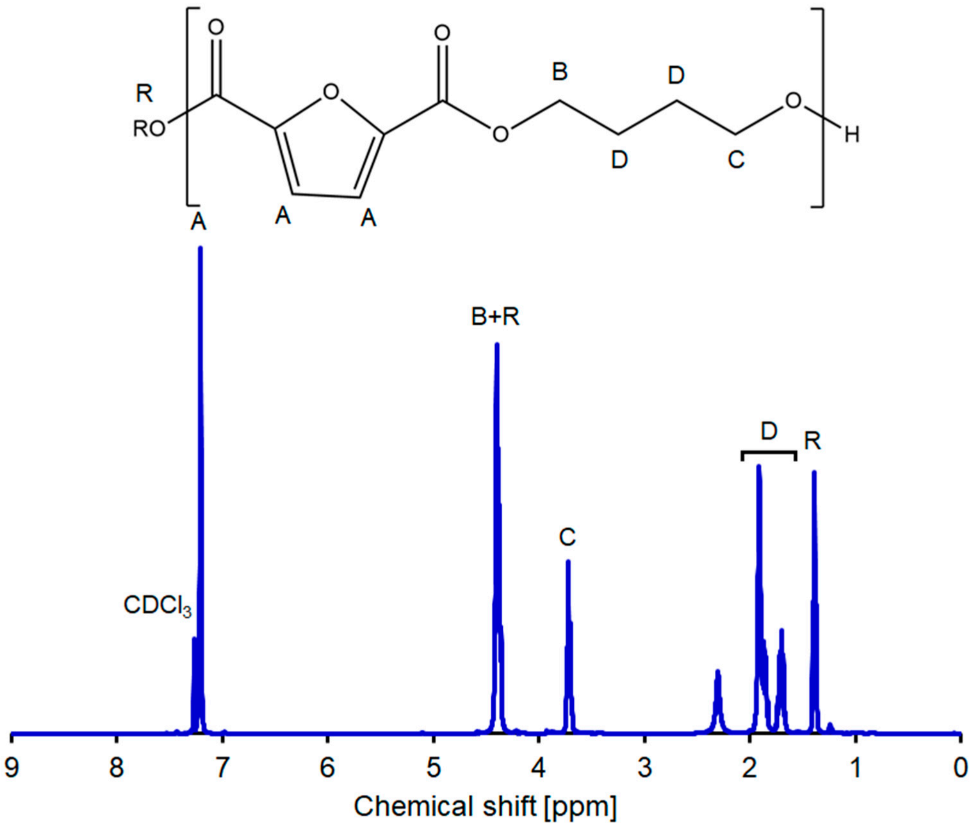

Figure S16. ^{1}H-NMR analysis of poly(1,4-butylene 2,5-furanoate) after the initial, solventless enzymatic synthesis step.

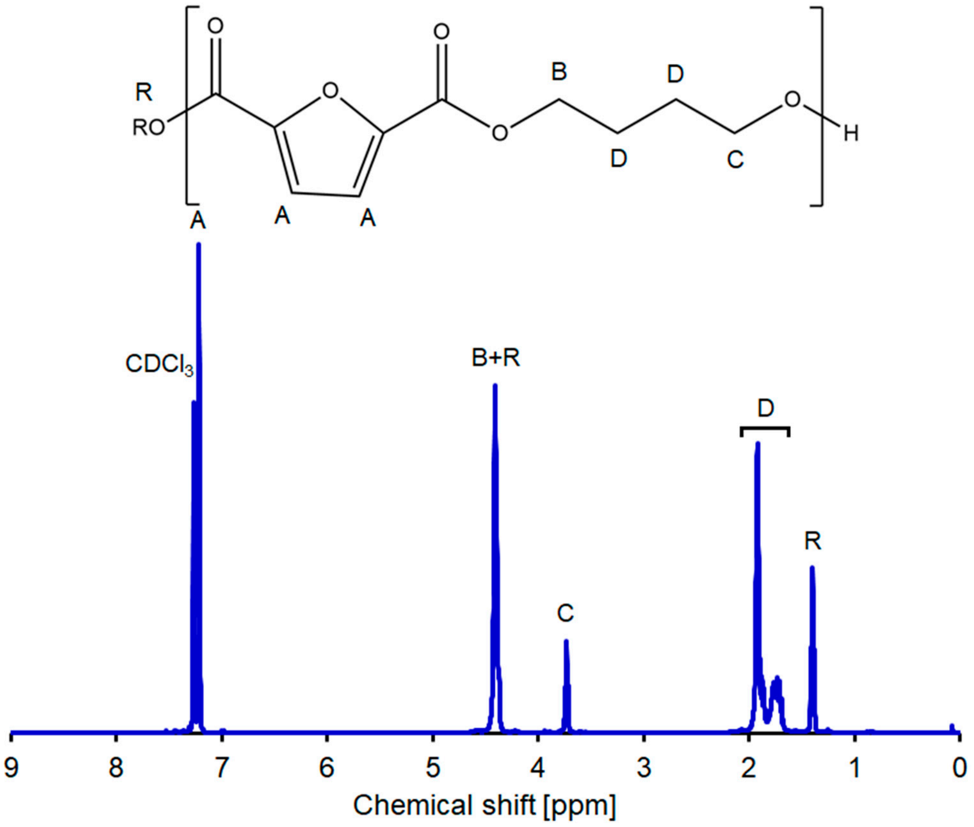

Figure S17. ^{1}H-NMR analysis of poly(1,4-butylene 2,5-furanoate) after the thermal upgrade conducted at 150 °C under vacuum.

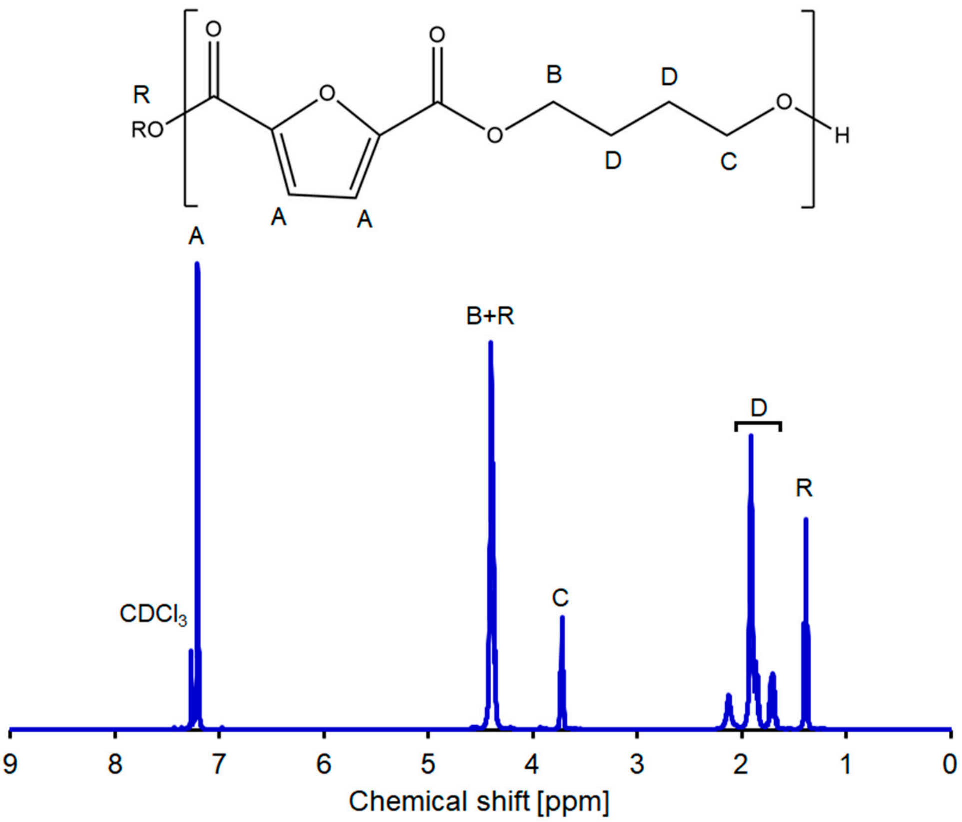

Figure S18. ^{1}H-NMR analysis of poly(1,4-butylene 2,5-furanoate) after the thermal upgrade conducted at 140 °C under air.

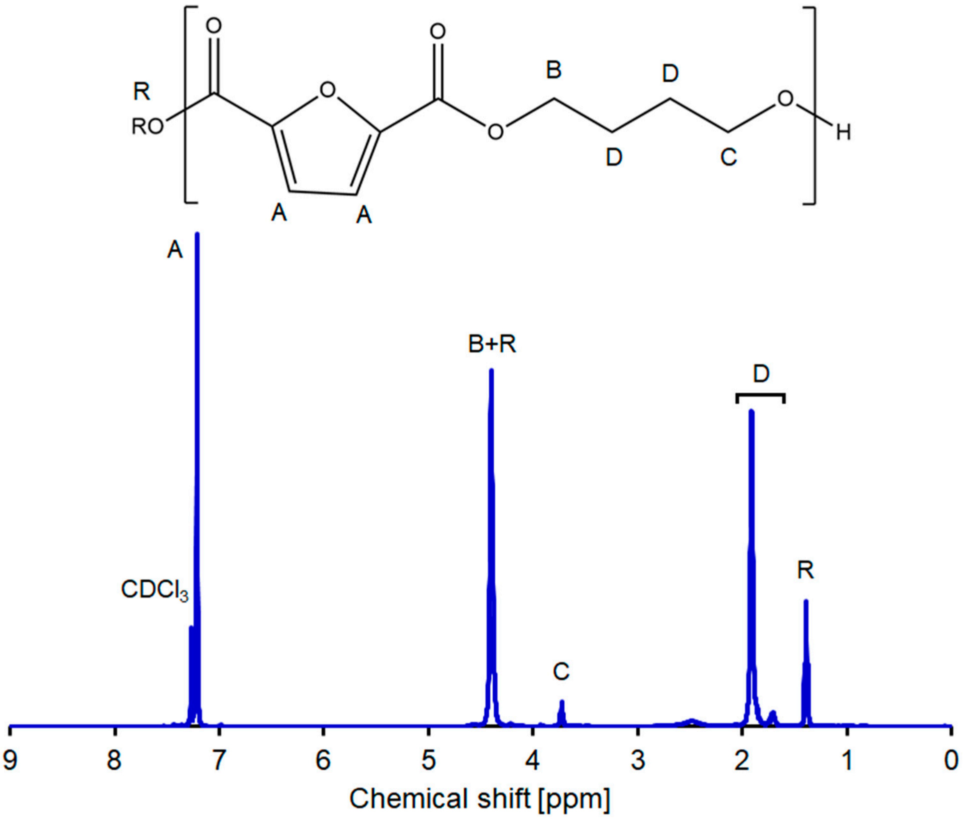

Figure S19. ^{1}H-NMR analysis of poly(1,4-butylene 2,5-furanoate) after the thermal upgrade conducted at 160 °C under air.

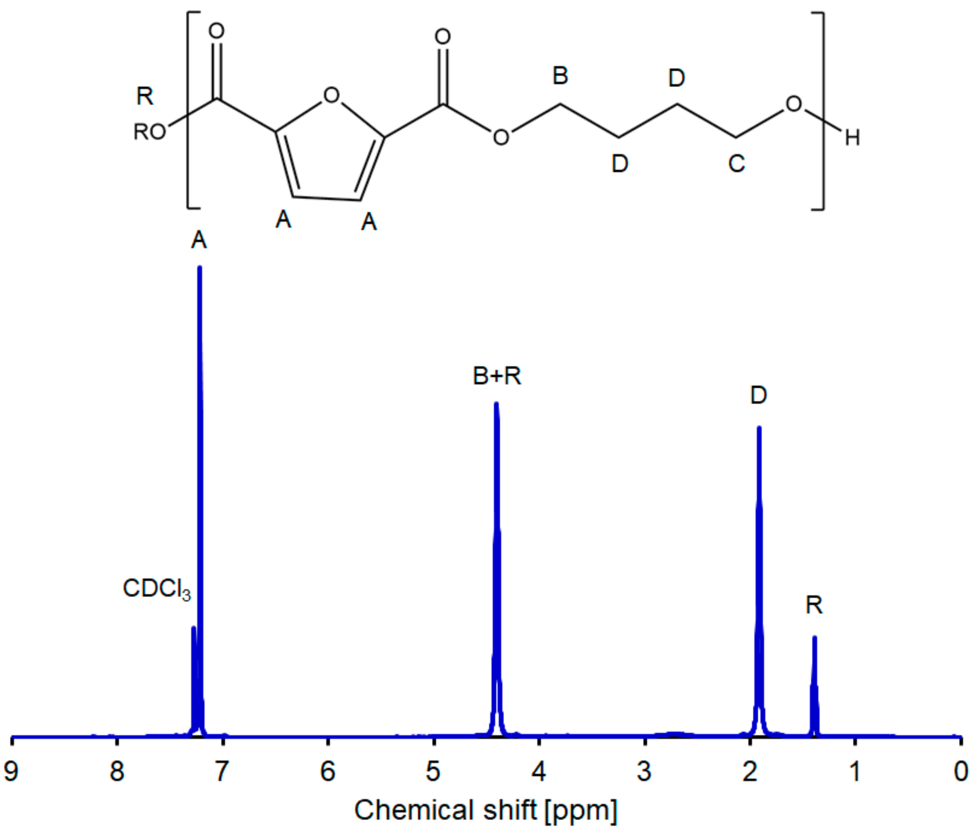

Figure S20. ^{1}H-NMR analysis of poly(1,4-butylene 2,5-furanoate) after the thermal upgrade conducted at 180 °C under air.

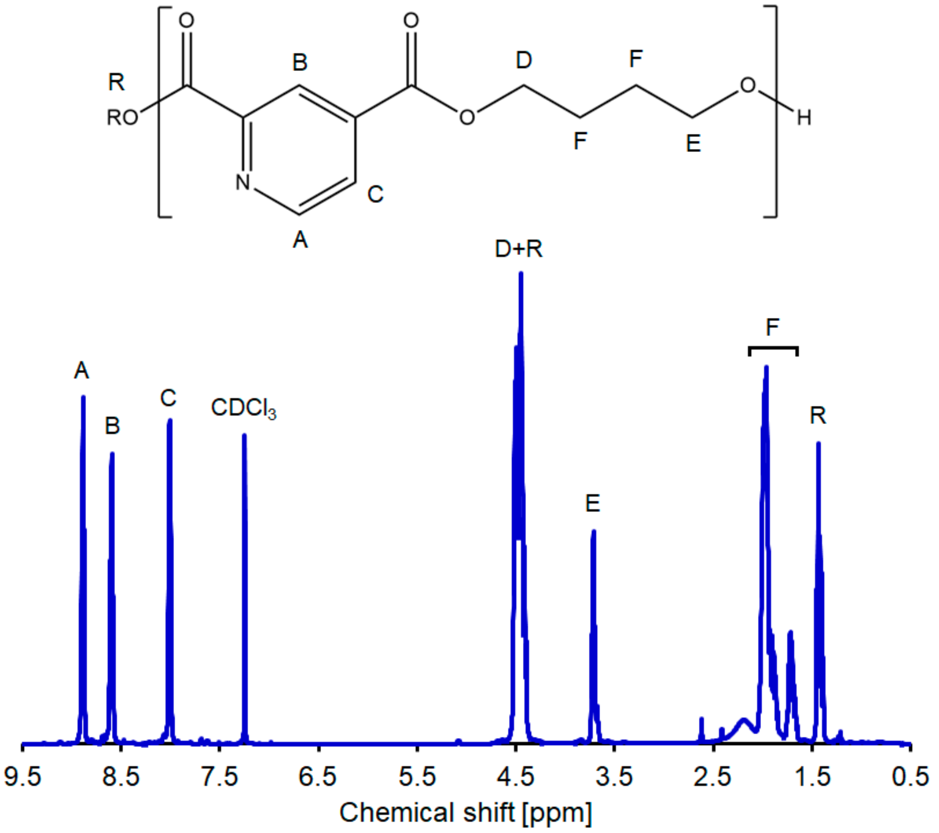

Figure S21. ^{1}H-NMR analysis of poly(1,4-butylene 2,4-pyridinedicarboxylate) after the initial, solventless enzymatic synthesis step.

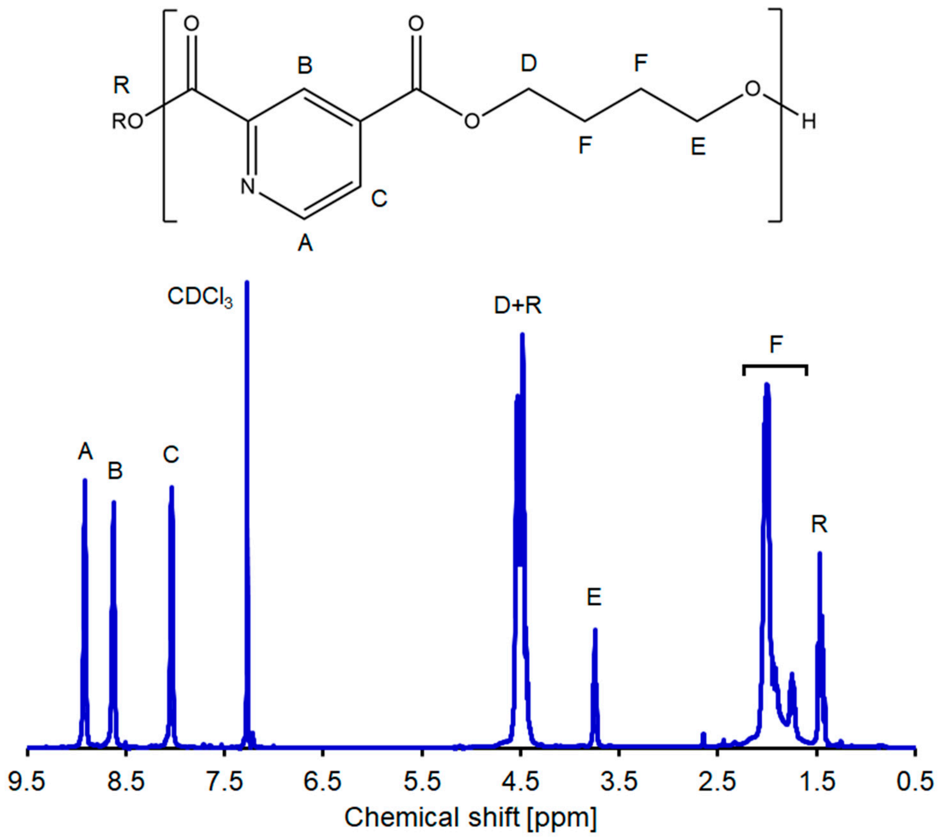

Figure S22. ^{1}H-NMR analysis of poly(1,4-butylene 2,4-pyridinedicarboxylate) after the thermal upgrade conducted at 150 °C under vacuum.

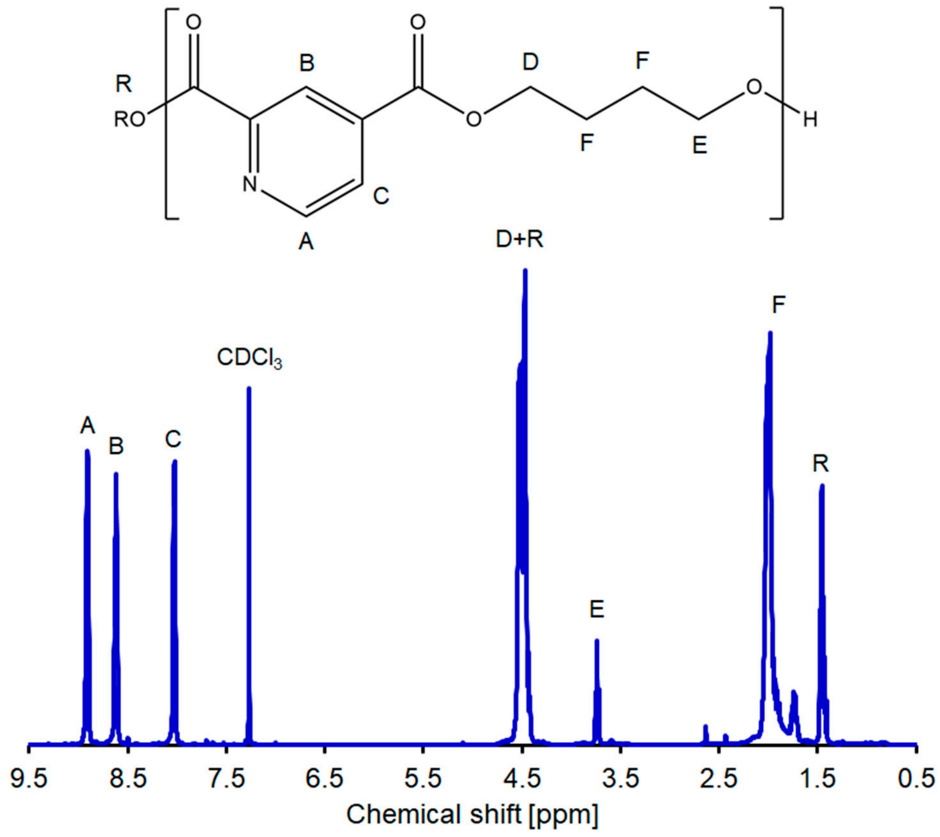

Figure S23. ^{1}H-NMR analysis of poly(1,4-butylene 2,4-pyridinedicarboxylate) after the thermal upgrade conducted at 140 °C under air.

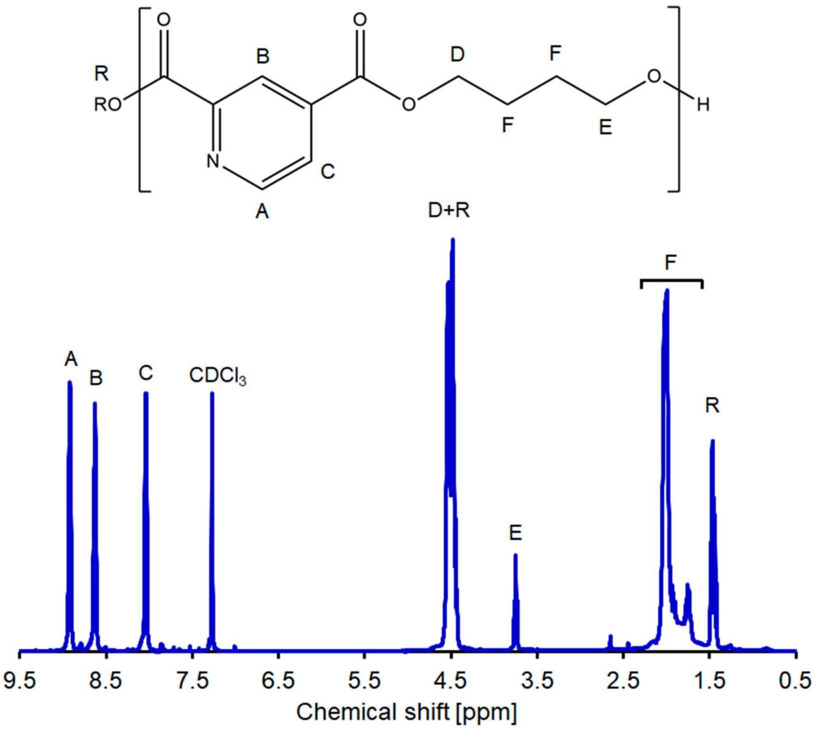

Figure S24. ^{1}H-NMR analysis of poly(1,4-butylene 2,4-pyridinedicarboxylate) after the thermal upgrade conducted at 160 °C under air.

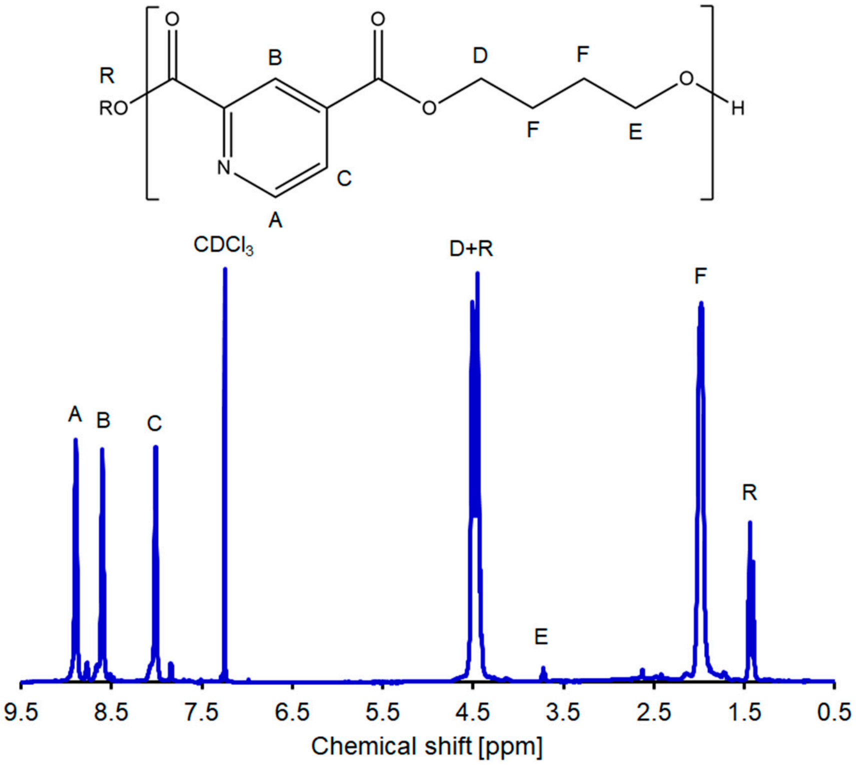

Figure S25. ^{1}H-NMR analysis of poly(1,4-butylene 2,4-pyridinedicarboxylate) after the thermal upgrade conducted at 180 °C under air.

© 2020 by the authors. Submitted for possible open access publication under the terms and conditions of the Creative Commons Attribution (CC BY) license (http://creativecommons.org/licenses/by/4.0/).
